# Micronutrient Status in Patients with Short Bowel Syndrome Weaned off Parenteral Support

**DOI:** 10.3390/nu17091598

**Published:** 2025-05-06

**Authors:** Nastasia Mattio, Charlotte Juin, Madeleine Lauverjat, Cécile Chambrier, Charlotte Bergoin, Thomas Couronne

**Affiliations:** Hépato-Gastroentérologie et Assistance Nutritionnelle, Hôpital Lyon Sud, 165 Chemin du Grand Revoyet, 69495 Pierre Bénite CEDEX, France

**Keywords:** micronutrients, short bowel syndrome, parenteral nutrition, intestinal failure, vitamins, trace elements

## Abstract

**Background/Objectives**: In short bowel syndrome adults (SBS), about 50% of patients on parenteral support (PS) are weaned off. However, micronutrient deficiency prevalence has never been studied in weaned patients. We aimed to assess the prevalence of micronutrient depletions and potential risk factors, more than a year after PS weaning. **Methods**: A retrospective study was conducted on our 161 weaned SBS patients between January 2011 and December 2021. Only 42 of them had an assessment of their plasma micronutrient status more than a year after PS weaning. **Results**: 40/42 patients had at least one micronutrient depletion, with an average of 4.0 ± 1.9 depletions per patient. Depletions in vitamins D, E, selenium, copper, and zinc were the most frequent, present for >50% of patients. In 75% of cases, patients depleted in vitamin B12 and D were already supplemented. Long-term proton pump inhibitor (PPI) use and duration on PS was associated with ≥ three depletions (*p* = 0.02). A daily mean of the total energy infused per week > 20 kcal/kg/day at the time of weaning was associated with more depletions (5.8 ± 1.6 vs. 3.7 ± 1.7 depletions, *p* = 0.02). Other factors (digestive anatomy, particularly SBS type, or associated chronic disease) were not predictive of depletion. **Conclusions**: Nearly all patients with SBS have at least one micronutrient depletion after PS withdrawal. This study suggests that a long-term monitoring of micronutrients status is needed for SBS patients weaned off PS. A larger-scale study would be necessary to generalize these results.

## 1. Introduction

Short bowel syndrome (SBS) is defined as a small intestine length of less than 2 m from the Treitz ligament, following an intestinal resection. It is a rare disease, affecting 5 to 80 patients per 1,000,000 in Europe [1,2]. The main etiologies include: mesenteric ischemia, inflammatory bowel diseases (IBD), and surgical complications. Extensive digestive resection often leads to malabsorption, resulting in at least a temporary dependence on parenteral nutrition (PN). In the context of SBS, it is estimated that 50% of patients will eventually be weaned off parenteral support (PS) [3]. Predictive factors for a successful weaning include: residual small bowel length, SBS type 2 or 3, presence of part of the ileum, hyperphagia, absence of underlying disease affecting the small bowel, and citrullinemia greater than 19 µmol/L at 2 years from resection [4,5].

Although caloric dependence is resolved after weaning from PS, the prevalence of micronutrient deficiencies in adults remains unknown. Micronutrients are essential components for human health. Their absorption is challenging and requires specific segments from the small bowel [6,7,8]. Table 1 summarizes the main absorption sites for each micronutrient. Due to intestinal resection, SBS patients weaned from PS are at high risk of micronutrient deficiencies. Micronutrient deficiencies can lead to severe clinical consequences if left untreated. For instance, these consequences may include Wernicke–Korsakoff encephalopathy for vitamin B1, scurvy for vitamin C, or Keshan cardiomyopathy for selenium [9,10].

In SBS children, micronutrient depletions have already been described after weaning from PS. These depletions affect up to 87.5% of weaned-off children. They primarily involve vitamin B12 and fat-soluble vitamins [11,12,13,14]. To our knowledge, no study has explored micronutrient status after weaning from PN in adults, except for a 2017 study that focused solely on vitamin D deficiency [15]. Micronutrients monitoring for patients under PS is well defined by recommendations established by various scientific societies [16,17,18,19]. Yet, no recommendation is made concerning patients weaned off PS, even though ESPEN underlines the risk of deficiency for these patients [17]. Caregivers or patients themselves unfortunately limit their clinical follow-up by considering “that they are cured”. However, clinical manifestations of micronutrient deficiencies can often occur after several months or even years. Since micronutrient deficiencies can have severe consequences on health, we decided to explore micronutrient depletions in adult SBS patients after PS withdrawal. The primary objective of our study was to determine the prevalence of micronutrient depletions (vitamins and trace elements) in SBS patients at least one year after PS withdrawal. We also aimed to identify potential risk factors associated with these depletions in this population.

## 2. Materials and Methods

### 2.1. Study Population and Follow-Up

This retrospective study was conducted on a longitudinal cohort of 821 patients who received PS between January 2011 and January 2021 (10 years) in the intensive clinical nutrition (ICN) unit (Lyon, France), a tertiary center specialized in intestinal failure.

To be included in this study, patients had to have SBS, defined as a small intestine length of less than 2 m. They had to be weaned off PS for more than a year and have undergone a plasma evaluation of micronutrient status between 1 and 5 years after withdrawal of PS.

**Figure 1:**
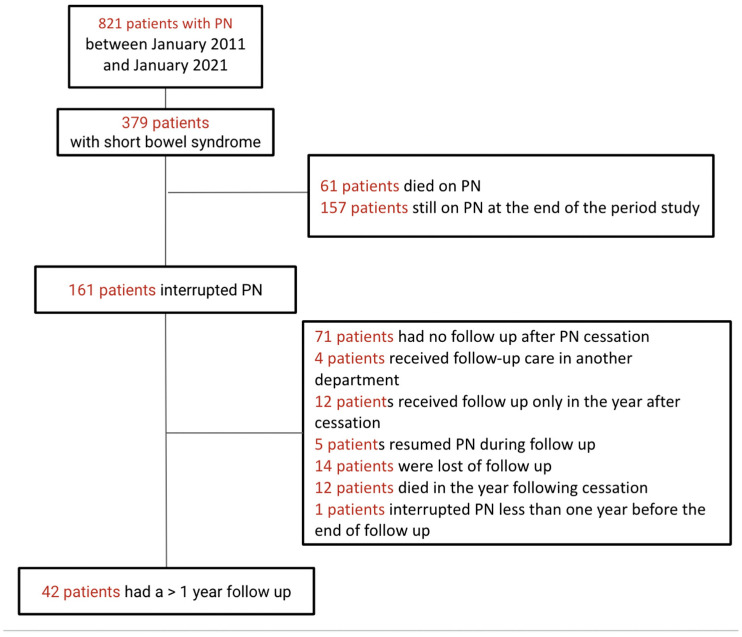
Flow chart.

Of the 379 patients with SBS followed in the center, 161 patients (42.4%) were able to discontinue PS. Among them, only 42 patients were followed for more than a year after discontinuation and had a reassessment that included micronutrient testing. For patients who underwent several micronutrients evaluations between 1 and 5 years after stopping PS, only the latest evaluation was considered. Patients who resumed PS within a year after stopping were not included in the study (Figure 1). Of the remaining 218 SBS patients, 61 (16.2%) died while on PS, and 157 (41.4%) were still on PS at the end of the inclusion period (Figure 1).

The study was approved by the Commision Nationale de l’Informatique et des Libertés (CNIL) and the ethics committee of Hospices Civils de Lyon on 17 May 2024 (number 24-5050). Data collection was conducted retrospectively from May to July 2024.

### 2.2. Data Collection

The description of the remaining bowel anatomy was recorded from surgical reports, anatomopathological reports and radiology when necessary. SBS type has been defined according to ESPEN guidelines [1]. The etiology of SBS, anthropometric measurements, medical history, and treatments were recorded by an experienced physician during successive consultations. The medical interview was conducted at the start of PS, at its discontinuation, and during the evaluation between 1 and 5 years after weaning. Anthropometric data were collected from hospitalization or consultation reports, while biological data were obtained from the center’s laboratory reports.

### 2.3. Measurements

Anthropometric parameters [triceps skinfold thickness, mid-upper arm circumference, muscle strength, weight, height, and body mass index (BMI)] were measured during the initial hospitalization and subsequent consultations. The risk of sarcopenia was defined, according to the French guidelines, by a muscle strength of less than 16 kPa in women and less than 26 kPa in men [20]. The severity of chronic intestinal failure definition was based on the daily mean of the total volume or energy infused per week at the time of weaning, according to ESPEN’s clinical classification [21].

Blood samples were taken at our hospital, during consultation, or in an outpatient clinic to assess micronutrient status (vitamins A, B1, B2, B3, B6, B9, B12, C, D, E, K1, zinc, copper, and selenium). These tests were conducted at the time of weaning and 1 to 5 years after discontinuation of PS. The laboratory technique and reference ranges used are reported in Table 2. In this study, according to the ESPEN micronutrient guideline, we assessed depletion of micronutrients. Depletion is defined by blood or plasma concentrations below the reference range, whereas deficiency is associated with clinical symptoms or metabolic disturbances [9,10]. Iron status wasn’t analyzed in our study, because of potential confounding factors.

### 2.4. Statistical Analysis

Categorical variables are described as numbers and percentages, and continuous variables as mean and standard deviation unless otherwise specified. For categorical variables, comparisons between groups were made using the Chi-square test or Fisher’s exact test as appropriate. For continuous variables, comparisons between groups were made using a *t*-test if the distribution was normal and the sample size in each group was >30, or using the non-parametric Mann–Whitney test. Normality was tested using the Shapiro–Wilk test. A *p*-value < 0.05 was considered statistically significant. All analyses were performed using R software version 3.5.3 (R Foundation for Statistical Computing, Vienna, Austria).

## 3. Results

### 3.1. Clinical Characteristics

Our cohort of 42 patients included 40.5% male patients, with a mean age of 62 ± 18 years at initiation of PS. The majority were diagnosed with type II SBS (64% at the time of the last evaluation). The evaluation took place on average 32 ± 17 months after discontinuation of PS. The two main etiologies of SBS were mesenteric ischemia (40.4%) and surgical complications (26.6%). General characteristics of the patients at the time of PS withdrawal are presented in Table 3. Mean duration on PS before withdrawal was significantly longer for patients with Crohn’s disease compared to those with other etiologies (87 ± 51 months vs. 56 ± 36 months, *p* = 0.04).

### 3.2. Nutritional Characteristics at Weaning

Before PS weaning, patients received an average of 4105 ± 3202 kcal and 5500 ± 588 mL per week as PS, distributed over 3.3 days. The average duration of PS before withdrawal was 61 ± 40 months.

The reasons for discontinuation were: restoration of bowel continuity (n = 20/42); intestinal adaptation (n = 17/42); catheter removal due to infection (n = 3/42). One patient requested to discontinue PS, and another had to stop infusions due to heart failure. Restoration of bowel continuity was performed on average 15 ± 19 months after initiation of PS. (Table 4).

### 3.3. Nutritional Characteristics

Almost all patients (40/42) were receiving at least one micronutrient supplementation after PS discontinuation. The most common supplements were vitamin D and B12, with 83% and 81% of patients receiving them, respectively. Among patients receiving vitamin B12, 20 (59%) were administered as intramuscular injections, while the others received oral supplementation. Trace elements were supplemented only for seven patients, either alone or as a micronutrient mixture (Table 5).

At nutritional reassessment, only one patient returned to their baseline weight, and two patients had a weight above their ideal weight. On average, patients lost 3.0 ± 9.3 kg between PS discontinuation and the follow-up consultation, representing about 4.4 ± 9.0% of their body weight. Fifteen patients (36%) lost more than 10% of their body weight since discontinuing PS, but the average BMI of this group was 22.0 kg/m^2^.

Regarding anthropometric parameters, complete data (both at PS weaning and at reassessment) were available for 32 patients. Triceps skinfold thickness decreased by an average of 5.7 mm (±7.8), or 20.7%. Arm circumference increased by an average of 1.5 cm (±2.8), and muscle strength by 2.7 kW (±7.8). At the time of reassessment, seven patients showed signs of sarcopenia, with PS resumption being recommended for two of them.

During nutritional reassessment, on an average of 32 months after the discontinuation of PS, there was an indication to resume PS in 9/42 patients. Four of them accepted it.

### 3.4. Micronutrient Depletions (Vitamins and Trace Elements)

In our study, 40 out of 42 (95%) patients had at least one micronutrient depletion, with an average of 4.0 ± 1.9 depletions per patient. Among them, 36 patients (86%) had at least one vitamin depletion, with an average of 2.4 ± 1.5 vitamin depletions per patient. Patients who had no vitamin depletions were all supplemented with vitamin D, and two were supplemented with B12. One was also supplemented with folates. Thirty-five patients (83%) had at least one trace element depletion, with an average of 1.5 ± 0.9 trace element depletions (Table 6).

**Figure 2:**
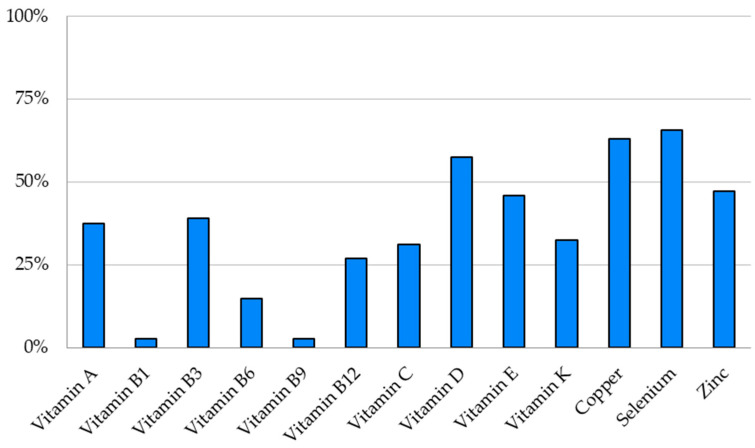
Proportion of patients with a micronutrient depletion, according to the type of micronutrient.

The most common depletion for vitamins was vitamin D (n = 23/40). Only two patients had an isolated vitamin D depletion. (Figure 2)

No patient had a vitamin B2 depletion, and only one had a vitamin B1 or B9 depletion. Thirty-three patients (79%) had at least one fat-soluble vitamin depletion (A, D, E, K).

More than 75% of patients with vitamin B12 and vitamin D depletions were already supplemented. Of the 34 patients supplemented with B12, 20 received intramuscular (IM) injections (15 of whom received a monthly 1 mg injection). There were four patients in this group with a depletion (20%). Fourteen patients were supplemented orally, 11 of whom on at least a weekly basis. Five patients were depleted in this group (45%). Among the 35 patients supplemented with vitamin D, only 4 received intramuscular injections (none were deficient in this group). Oral supplementation protocols were highly heterogeneous.

None of the patients with B3, B6, or B9 depletions were supplemented.

Concerning trace elements, depletions were frequent in our cohort (47–65%) (Table 4). The most common depletion for trace elements was selenium (65%), followed closely by copper depletion (Figure 2). Only a few patients were already supplemented, less than 15%.

### 3.5. Micronutrient Depletions According to SBS Characteristics

**Figure 3:**
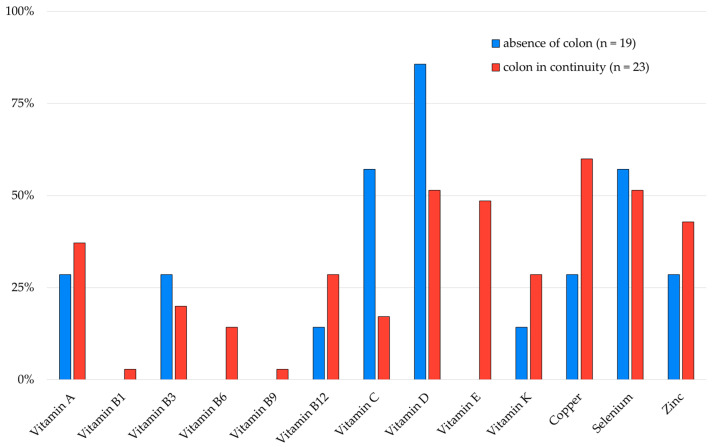
Proportion of micronutrient depletions based on the presence or absence of a colon in continuity.

Patients without colon (n = 4) had an average of 3.1 ± 1.5 micronutrient depletions, compared to an average of 4.1 ± 1.9 for those with a preserved colon (n = 38). Small bowel length was 128.6 ± 51.5 cm in the former group, versus 105.3 ± 35.9 cm in the latter. More than half of the patients with type I SBS had depletions in vitamin C, vitamin D, and/or selenium. If the colon was in continuity, more than half of the patients exhibited depletions in vitamin D, copper, and selenium (Figure 3).

### 3.6. Depletions’ Risk Factors (Table 7)

In univariate analysis, a daily mean of the total volume infused per week greater than 1 L at weaning tends to be associated with the highest number of depletions (5.1 ± 1.8 vs. 3.7 ± 1.8, *p* = 0.09). A daily mean of the total energy infused per week exceeding 20 kcal/kg/day before weaning off PS was significantly associated with a higher risk of depletion (5.8 ± 1.6 vs. 3.7 ± 1.7 kcal/kg/day, *p* = 0.02).

**Table 7:** Number of micronutrient depletions by type, etiology, and severity of short bowel syndrome (SBS) at withdrawal of parenteral support (PS).

SBS Characteristics	Number of Depletions (mean +/− SD)
*SBS type*	
Type 1	3.1 ± 1.5
Type 2	4.0 ± 2.0
Type 3	4.4 ± 1.8
*SBS etiology*	
IBD	3.5 ± 1.9
Mesenteric ischemia	3.7 ± 2.1
Surgical complications	4.1 ± 2.5
Other	4.2 ± 0.9
*Severity of intestinal failure at weaning based on the daily mean of the total volume infused per week* *	
<1 L/day	3.7 ± 1.8
1–2 L/day	4.5 ± 2.1
2–3 L/day	4.3 ± 2.3
>3 L/day	6.5 ± 0.7
*Severity of intestinal failure at weaning based on the daily mean of the total kcal infused per week, adjusted on weight* **	
<1 kcal/kg/day	4.0 ± 0
1–10 kcal/kg/day	3.8 ± 1.8
11–20 kcal/kg/day	3.5 ± 1.6
>20 kcal/kg/day	5.8 ± 1.6

* = (volume infused per day) × (number of infusion per week)/7. ** = [(kcal infused per day) × (number of infusion per week)/7]/weight of the patient.

Long-term use of proton pump inhibitors (PPIs) (22/42 patients) was associated with ≥ three depletions (86% vs. 65% patients, *p* = 0.02). Similarly, prolonged PS before weaning was associated with ≥ 3 depletions (*p* = 0.04), with a mean PS duration of 52 months for patients with < 3 depletions and a mean duration of 87 months for the others.

In contrast, having a colon in continuity (4.1 ± 1.9 depletions vs. 3.1 ± 1.5, *p* = 0.16), chronic kidney disease (13/42 patients, 4.3 ± 1.0 depletions vs. 3.9 ± 2.0, *p* = 0.30), IBD (6/42 patients, 3.5 ± 2.0 depletions vs. 4.0 ± 1.8, *p* = 0.56), sarcopenia (7/42 patients, 3.1 ± 1.3 depletions vs. 4.1 ± 1.9, *p* = 0.13), or a weight loss >10% after weaning (16/42 patients, 4.0 ± 1.8 depletions vs. 3.9 ± 1.9, *p* = 0.68) were not associated with a higher number of depletions.

## 4. Discussion

This retrospective study reveals that micronutrient depletions are frequent in the adult SBS population, even long after withdrawal of PS. To our knowledge, this is the first study conducted in adults with SBS that describes micronutrient depletions after prolonged PS withdrawal. We observed that 95% of patients had at least one micronutrient depletion, with an average of four depletions per patient. Depletions in vitamins and trace elements affected 85% and 86% of patients, respectively. These results are significantly higher than those reported by G.M. Van Der Werf et al., who identified trace element deficiencies in 41% of 110 patients with chronic intestinal insufficiency, but still on PS. [22]

Our results are comparable to those obtained in pediatric studies, where vitamin deficiencies were observed in 87.5% of weaned patients, a result very close to ours (87.6%). Fat-soluble vitamin deficiencies and vitamin B12 deficiencies were the most frequently reported in the pediatric population. In our study, fat-soluble vitamin depletion was frequent (79%). But vitamin B12 depletion was lower than in the pediatric study (27%). This may be explained by a better supplementation, as more than 75% of our patients were already supplemented for vitamin B12. Trace elements depletion was frequent in our study (83%), which seems to be a new datum, as trace elements were poorly studied in pediatric studies. Weaning from PS is, however, faster in the pediatric population (10.8 months vs. 61 months in our study) [11,23]. H. Feng et al. also emphasized the need for long-term supplementation after PN weaning, with deficiencies appearing as early as the first month after PS withdrawal.

The most common vitamin depletions in our study were vitamin D and vitamin E. The absorption of these fat-soluble vitamins is limited in SBS due to the frequent dysfunction of the enterohepatic bile acid cycle associated with ileal resection. Bacterial overgrowth induced by surgeries involving the ileocecal valve and caecum also contributes to this malabsorption by causing inflammation and epithelial atrophy of the small intestinal mucosa [24].

More than half of patients with type 1 SBS had depletions in vitamin D, selenium, and/or vitamin C. This could be explained by the fact that among the seven affected patients, five no longer had an ileum with a jejunostomy. Ileum is the primary site for vitamin C absorption. In patients with a colon in continuity, more than half had depletions in vitamin D, selenium, and/or copper. In this group, nearly one-third of patients (12/36) had undergone a jejunal resection, the preferred site for copper absorption.

All patients had a CRP level below 25 mg/L, allowing the interpretation of micronutrient blood levels [9]. Interpretation of vitamin A concentration should ideally be based on Retinol Binding Protein (RBP) levels, and copper concentration should be interpreted based on ceruloplasmin levels. However, these data were only available for 16 and 17 patients, respectively, in our cohort. Therefore, we based our interpretation on vitamin A and copper blood levels alone. Notably, a low RBP (below 30 mg/L) was associated with a vitamin A depletion in only two patients. A low copper level was associated with normal ceruloplasmin (above 0.15 g/L) in four patients.

The interpretation of vitamin E blood concentration depends on cholesterol levels. Hypercholesterolemia, due to increased lipoproteins in the blood, can lead to an artificially high concentration of vitamin E. In our study, two patients had cholesterol levels above normal (5 mmol/L), one had a tocopherol concentration above normal (59.0 µmol/L), and the other had a normal concentration at 29.7 µmol/L. There was no hypocholesterolemia in our population [25].

Concerning fat-soluble vitamins, it would be interesting to evaluate their status based on steatorrhea to investigate if these vitamins’ status is correlated with fat malabsorption. A datum that wasn’t available in our study, unfortunately.

Finally, the interpretation of a serum zinc depletion should only be made in the presence of normal serum albumin, as 60% of serum zinc is bound to this protein [26]. In our cohort, only two patients with low zinc levels also had albumin levels below 35 g/L.

In our study, iron wasn’t analyzed due to potential confounding factors. Interpretation of iron status can be difficult due to the various etiologies for iron deficiency besides malabsorption [27]. Therefore, establishing a specific link between small bowel syndrome malabsorption and iron deficiency seemed difficult for a retrospective study.

Despite the high prevalence of depletions in our study, more than half of the patients were not supplemented with micronutrients (aside from vitamins D and B12), particularly trace elements (less than 15% of patients received supplementation despite an identified depletion). Concerning vitamins D and B12, respectively, 29% and 26% of long-term supplemented patients still had depletions. However, it seems difficult to distinguish a malabsorption from a non-compliance in a retrospective study. Concerning vitamin D, Fan et al. identified the length of the small intestine and the duration of SBS as risk factors for deficiency [15].

Supplementation doses and administration routes were heterogeneous among our cohort. Vitamin D supplementation seems more effective when administered intramuscularly on a monthly basis or orally on a weekly basis. Similarly, 20% of patients supplemented with vitamin B12 intramuscularly remained depleted, compared to 85% in those receiving oral supplementation. Although we cannot draw significant conclusions due to the limited size of our cohort, these observations align with other studies comparing supplementation efficacy [28]. A study on the most effective administration routes in patients with intestinal insufficiency would be pertinent. Furthermore, micronutrients supplementation can be challenging in some countries, especially in France, where these treatments aren’t covered by insurances.

No patient reported clinical consequences of these micronutrient depletions during consultations. However, symptoms can sometime be subclinical, and a prospective study would seem relevant to explore these clinical consequences. In this sense, the 2023 EPSEN guidelines on chronic intestinal insufficiency recommend annual micronutrient testing for patients with short bowel syndrome under PS [17].

SBS type didn’t seem predictive of depletions in our cohort. For type I SBS, this could be explained by a longer residual small bowel (50 cm more than patients with colon in continuity), potentially allowing more efficient micronutrient absorption. For type II and III SBS, absorption may have been facilitated by intestinal adaptation mechanisms, such as the development of colonic villi in number and length [29,30].

Long-term use of PPIs appeared to be associated with more depletions in our study. The link between these treatments and deficiencies, especially vitamin B12, is well documented [31,32]. Gastric acidity plays a crucial role in dissociating vitamin B12 from its ligand, facilitating its binding to intrinsic factor and ileal absorption. It seems essential to regularly assess the need to continue PPIs for patients with SBS.

Although our study did not include patients who had switched from PS to enteral nutrition (EN), it seems that these patients also require prolonged follow-up. Several studies have reported micronutrient deficiencies in SBS patients on prolonged EN or intermittent PS [12,13,33].

The PS content in terms of kcal and mL per week was high at the time of discontinuation (around 4105 kcal and 5500 mL per week). This was particularly true for patients who underwent continuity restoration; they received an average of 7700 mL and 5141 kcal per week, compared to 3400 mL and 3100 kcal per week for patients weaned without restoration.

In our initial cohort, 83 (52%) patients with SBS who were weaned off PS didn’t have a follow-up on micronutrient status after cessation. This isn’t an isolated case, as a 2016 ESPEN report highlighted a lack of follow-up, attributing it to practitioners’ lack of understanding of the issues related to the diagnosis and supplementation of micronutrient deficiencies [34].

Clearly, our study has several limitations. Firstly, it is a monocentric study, which may limit the generalization of our results and the cohort’s representativeness. Recruitment was carried out in a specialized center. A tertiary center specialized in intestinal failure may provide better follow-up, which may underestimate the prevalence of micronutrient depletions. Secondly, although SBS is a rare disease, the size of our cohort remains small. This implies two limits. First, a lack of statistical power that may limit our findings, especially concerning risk factors of micronutrient depletions. Additionally, the small sample size of our cohort may amplify a selection bias. In fact, we can assume that patients who pursued a follow-up in our center may be subject to more severe depletions, thus overestimating the prevalence of micronutrient depletions. Finally, our study was retrospective. Therefore, we couldn’t analyze the clinical consequences of micronutrient depletions in our cohort. This limits the clinical relevance of the study. However, our study opens the way for a new research area for SBS patients. And it would be relevant to complete these findings with a multicentric and prospective study that could generalize and specify these conclusions, given the rarity of this disease and its specialized management. Moreover, a larger-scale study could help identify associated or even predictive factors of micronutrient deficiencies. Such studies may help define recommendations on the follow-up of SBS patients weaned off PS. Until then, it would seem relevant to follow ESPEN recommendations for patients under HPN, with an annual evaluation of micronutrients status even after discontinuation of PS.

No patients treated with GLP-2 analogues were included in our study, as none had been weaned long enough to be eligible. To date, there is no study on micronutrient deficiencies for weaned patients on GLP-2 analogues.

It is noteworthy that none of the selected patients had radiation enteritis, although it accounts for 7% of SBS cases in Europe [21]. This may be explained by a poorer nutritional prognosis for this etiology, likely related to associated comorbidities. Additionally, alterations of the small bowel mucosa in this disease decreases the mucosal adaptation capacity, limiting the possibilities of weaning off PS for these patients [3].

## 5. Conclusions

In conclusion, micronutrient depletions are numerous and heterogeneous in patients with SBS after prolonged cessation of PS. Our results highlight the importance of long-term monitoring of this population, regardless of digestive anatomy. Weaning from PS does not guarantee adequate micronutrients absorption, and long-term monitoring of micronutrients seems essential for these patients. Further studies are needed to explore the clinical consequences of these depletions, and to define the follow-up for these patients.

## Figures and Tables

**Table 1:** Main absorption sites for each micronutrient.

Micronutrients	Absorption Sites
Vitamin A	Proximal jejunum
Vitamin E	Jejunum
Vitamin D	Distal jejunum and ileum
Vitamin K	Ileum
Vitamin B1	Proximal jejunum
Vitamin B3	Proximal jejunum
Vitamin B6	Proximal jejunum
Vitamin B9	Jejunum
Vitamin B12	Ileum
Vitamin C	Distal jejunum and ileum
Zinc	Jejunum
Copper	Stomach and duodenum
Selenium	Duodenum and proximal jejunum

**Table 2:** Micronutrients reference range.

Micronutrient	Laboratory Technique	Reference Range
Vitamin A	LC-UV on heparinized plasma	1.4–3.2 µmol/L
Vitamin B1	LC-FLD on whole blood	66–200 nmol/L
Vitamin B3	UPLC on whole blood	38–56 µmol/L
Vitamin B6	LC-FLD on whole blood	15–73 µmol/L
Vitamin B9	ECLIA on serum	7–39.6 µg/L
Vitamin B12	ECLIA on serum	187–974 ng/L
Vitamin C	LC-ECD on heparinized plasma	25–85 µmol/L
Vitamin D	ECLIA on serum	75–150 nmol/L
Vitamin E	LC-UV on heparinized plasma	20–35 µmol/L
Vitamin K	HPLC-MS on serum	100–1000 ng/L
Copper	ICP-OES on serum	14–19 µmol/L
Selenium	ICP-MS on serum	0.9–1.5 µmol/L
Zinc	ICP-OES on serum	9.4–15.2 µmol/L

LC-FLD: Liquid chromatography with fluorescence detection; LC-UV: Liquid chromatography with ultraviolet detection; UPLC: Ultra-high-performance liquid chromatography; ECLIA: Electrochemiluminescence immunoassay; LC-ECD: Liquid chromatography with electrochemical detection; HPLC-MS: High-performance liquid chromatography-mass spectrometry; ICP-OES: inductively coupled plasma optical emission spectroscopy; ICP-MS: Inductively coupled plasma mass spectrometry.

**Table 3:** General characteristics of patients at PS withdrawal (n = 42).

Characteristics	
Age (years) (mean +/− SD)	62 +/− 18
Gender, male, n (%)	17 (40.5%)
*SBS sub-type*, n (%)	
I	7 (17%)
II	27 (64%)
III	8 (19%)
*SBS etiology*, n (%)	
Mesenteric ischemia	17
Surgical complications	11
Inflammatory bowel diseases (IBD)	6
Oncology	3
Trauma	3
Radiation enteritis	0
Chronic intestinal pseudo-obstruction (CIPO)	0
Other (small bowel volvulus)	2
*Comorbidities*	
Hypertension	15 (36%)
Oncological history	9 (28%)
Obesity or history of obesity	9 (28%)
Chronic kidney disease	13 (31%)
Dyslipidemia	6 (19%)
Diabetes	4 (10%)
Heart failure	5 (12%)
Small bowel length in situ (cm) (mean +/− SD)	109 +/− 42
Percentage of colon in situ (%) (mean +/− SD)	75 +/− 31
History of enterocutaneous fistula, n (%)	6 (14%)
History of total colectomy, n (%)	4 (10%)
History of bowel continuity restoration, n (%)	23 (55%)
*Citrullinemia* (n = 16)	
Citrullinemia (μmol/L) (mean +/− SD)	29 +/− 123
Patients with citrullinemia < 20 μmol/L, n (%)	3 (19%)
Number of patients with kidney failure (GFR < 60 mL/min/1.73 m^2^), n (%)	13 (81%)

**Table 4:** Nutritional status and timelines during follow-up (n = 42).

Characteristics	
BMI with usual weight before SBS (kg/m^2^) (mean +/− SD)	27.0 +/− 7.6
BMI with weight at initiation of PS (kg/m^2^) (mean +/− SD)	23.4 +/− 6.6
BMI with weight at reassessment (kg/m^2^) (mean +/− SD)	24.6 +/− 6.4
Duration on PS before weaning off (months) [Med (IQR)]	45.5 (40.5)
*Severity of intestinal failure at weaning based on the daily mean of the total volume infused per week* *	
<1 L/day	35 (83%)
1–2 L/day	2 (5%)
2–3 L/day	3 (7%)
>3 L/day	2 (5%)
*Severity of intestinal failure at weaning based on the daily mean of the total kcal infused per week, adjusted on weight* **	
<1 kcal/kg/day	1 (2%)
1–10 kcal/kg/day	28 (67%)
11–20 kcal/kg/day	8 (19%)
>20 kcal/kg/day	5 (12%)
Serum albumin at reassessment (g/L) (mean +/− SD)	38.0 +/− 7.7
C-Reactive Protein (CRP) at reassessment (mg/L) (mean +/− SD)	6.6 +/− 1.1

* = (volume infused per day) × (number of infusion per week)/7. ** = [(kcal infused per day) × (number of infusion per week)/7]/weight of the patient.

**Table 5:** Ongoing micronutrient supplementation at reassessment (n = 42).

Ongoing Supplementation	Number of Patients
Vitamin D	35
Vitamin B12	34
Vitamin E	8
Vitamin K	8
Micronutrient mixture	6
Vitamin A	5
Vitamin C	4
Vitamin B9	3
Vitamin B6	2
Vitamin B1	1
Selenium	1

**Table 6:** Micronutrient depletions and the number of patients with a depletion per micronutrient.

Micronutrient	Patients with Depletion	Plasma Concentration of Micronutrient in Patients with Depletions Med (IQR)
*Fat-soluble vitamins*		
Vitamin A (µmol/L)	15/40	1.2 (0.4)
Vitamin E (µmol/L)	17/37	15.2 (5.1)
Vitamin K1 (ng/L)	11/34	83.0 (25.5)
Vitamin D (nmol/L)	23/40	48.0 (28. 3)
*Water-soluble vitamins*		
Vitamin B1 (nmol/L)	1/36	47.1 (0)
Vitamin B3 (µmol/L)	9/23	34.0 (8.0)
Vitamin B6 (nmol/L)	5/34	7 (0.9)
Vitamin B9 (µg/L)	1/36	1.5 (0)
Vitamin B12 (ng/L)	11/41	131.0 (48.0)
Vitamin C (µmol/L)	10/32	14.0 (5.8)
*Trace elements*		
Copper (µmol/L)	22/35	10.7 (3.6)
Selenium (µmol/L)	23/35	0.7 (0.2)
Zinc (µmol/L)	17/36	8.3 (1.7)

## Data Availability

The original contributions presented in this study are included in the article. Further inquiries can be directed to the corresponding author(s).

## Abbreviations

The following abbreviations are used in this manuscript:

SBS	Short bowel syndrome
IBD	Inflammatory bowel disease
PN	Parenteral nutrition
PS	Parenteral support
ICN	Intensive clinical nutrition
BMI	Body mass index
CRP	C-reactive protein
IM	Intramuscular
PPIs	Proton pump inhibitors
RBP	Retinol binding protein
EN	Enteral nutrition

## References

[1] Pironi L. (2023). Definition, classification, and causes of short bowel syndrome. Nutr. Clin. Prac..

[2] Brandt C.F., Hvistendahl M., Naimi R.M., Tribler S., Staun M., Brøbech P., Jeppesen P.B. (2017). Home Parenteral Nutrition in Adult Patients With Chronic Intestinal Failure: The Evolution Over 4 Decades in a Tertiary Referral Center. J. Parenter. Enter. Nutr..

[3] Pironi L., Goulet O., Buchman A., Messing B., Gabe S., Candusso M., Bond G., Gupte G., Pertkiewicz M., Steiger E. (2012). Outcome on home parenteral nutrition for benign intestinal failure: A review of the literature and benchmarking with the European prospective survey of ESPEN. Clin. Nutr..

[4] Crenn P., Coudray–Lucas C., Thuillier F., Cynober L., Messing B. (2000). Postabsorptive plasma citrulline concentration is a marker of absorptive enterocyte mass and intestinal failure in humans. Gastroenterology.

[5] Amiot A., Messing B., Corcos O., Panis Y., Joly F. (2013). Determinants of home parenteral nutrition dependence and survival of 268 patients with non-malignant short bowel syndrome. Clin. Nutr..

[6] Reboul E. (2011). Absorption intestinale des vitamines liposolubles. OCL.

[7] Bonnefond-Ortega M., Goudable J., Chambrier C., Bétry C. (2018). L’absorption intestinale des vitamines hydrosolubles et liposolubles en pratique clinique. Nutr. Clin. Métabolisme.

[8] Reboul E. (2017). Vitamin E Bioavailability: Mechanisms of Intestinal Absorption in the Spotlight. Antioxidants.

[9] Berger M.M., Shenkin A., Schweinlin A., Amrein K., Augsburger M., Biesalski H.K., Bischoff S.C., Casaer M.P., Gundogan K., Lepp H.-L. (2022). ESPEN micronutrient guideline. Clin. Nutr..

[10] Berger M.M., Shenkin A., Schweinlin A., Amrein K., Augsburger M., Biesalski H.K., Bischoff S.C., Casaer M.P., Gundogan K., Lepp H.L. (2024). Corrigendum to “ESPEN micronutrient guideline” [Clin Nutr 41 (2022) 1357–1424/YCLNU5151]. Clin. Nutr..

[11] Chatzidaki V., Wood R., Alegakis A., Lawson M., Fagbemi A. (2023). Parenteral support and micronutrient deficiencies in children with short bowel syndrome: A comprehensive retrospective study. Clin. Nutr. ESPEN.

[12] Ubesie A.C., Kocoshis S.A., Mezoff A.G., Henderson C.J., Helmrath M.A., Cole C.R. (2013). Multiple Micronutrient Deficiencies among Patients with Intestinal Failure during and after Transition to Enteral Nutrition. J. Pediatr..

[13] Yang C.F., Duro D., Zurakowski D., Lee M., Jaksic T., Duggan C. (2011). High Prevalence of Multiple Micronutrient Deficiencies in Children with Intestinal Failure: A Longitudinal Study. J. Pediatr..

[14] Feng H., Zhang T., Yan W., Lu L., Tao Y., Cai W., Wang Y. (2020). Micronutrient deficiencies in pediatric short bowel syndrome: A 10-year review from an intestinal rehabilitation center in China. Pediatr. Surg. Int..

[15] Fan S., Ni X., Wang J., Zhang Y., Tao S., Kong W., Li Y., Li J. (2017). High Prevalence of Suboptimal Vitamin D Status and Bone Loss in Adult Short Bowel Syndrome Even After Weaning Off Parenteral Nutrition. Nutr. Clin. Prac..

[16] Mercer-Smith G.W., Kirk C., Gemmell L., Mountford C., Nightingale J., Thompson N. (2021). British Intestinal Failure Alliance (BIFA) guidance—Haematological and biochemical monitoring of adult patients receiving home parenteral nutrition. Frontline Gastroenterol..

[17] Pironi L., Cuerda C., Jeppesen P.B., Joly F., Jonkers C., Krznarić Ž., Lal S., Lamprecht G., Lichota M., Mundi M.S. (2023). ESPEN guideline on chronic intestinal failure in adults—Update 2023. Clin. Nutr..

[18] Osland E.J., Ali A., Nguyen T., Davis M., Gillanders L. (2016). Australasian society for parenteral and enteral nutrition (AuSPEN) adult vitamin guidelines for parenteral nutrition. Asia Pac. J. Clin. Nutr..

[19] Siepler J. (2007). Principles and Strategies for Monitoring Home Parenteral Nutrition. Nutr. Clin. Prac..

[20] HAS: Diagnostic de la dénutrition chez l’enfant, l’adulte, et la personne de 70 ans et plus. https://www.has-sante.fr/upload/docs/application/pdf/2021-11/reco368_fiche_outil_denutrition_pa_cd_20211110_v1.pdf.

[21] Pironi L., Konrad D., Brandt C., Joly F., Wanten G., Agostini F., Chambrier C., Aimasso U., Zeraschi S., Kelly D. (2018). Clinical classification of adult patients with chronic intestinal failure due to benign disease: An international multicenter cross-sectional survey. Clin. Nutr..

[22] Van Der Werf G.M., Van Rijssen N.M., Jonkers C.F., Boermeester M.A., Serlie M.J. (2023). Trace-Element Status In Patients With Intestinal Failure Type II And III. Clin. Nutr. ESPEN.

[23] Wu J., Tang Q., Feng Y., Huang J., Tao Y., Wang Y., Cai W., Shi C. (2007). Nutrition assessment in children with short bowel syndrome weaned off parenteral nutrition: A long-term follow-up study. J. Pediatr. Surg..

[24] Cole C.R., Ziegler T.R. (2007). Small bowel bacterial overgrowth: A negative factor in gut adaptation in pediatric SBS. Curr. Gastroenterol. Rep..

[25] Thurnham D.I., Davies J.A., Crump B.J., Situnayake R.D., Davis M. (1986). The Use of Different Lipids to Express Serum Tocopherol: Lipid Ratios for the Measurement of Vitamin E Status. Ann. Clin. Biochem..

[26] Jeejeebhoy K.N. (2002). Short bowel syndrome: A nutritional and medical approach. CMAJ.

[27] Kumar A., Sharma E., Marley A., Samaan M.A., Brookes M.J. (2022). Iron deficiency anaemia: Pathophysiology, assessment, practical management. BMJ Open Gastroenterol..

[28] Tafaro L., Nati G., Leoni E., Baldini R., Cattaruzza M.S., Mei M., Falaschi P. (2013). Adherence to anti-osteoporotic therapies: Role and determinants of “spot therapy”. Osteoporos. Int..

[29] Haastrup P.F., Thompson W., Søndergaard J., Jarbøl D.E. (2018). Side Effects of Long-Term Proton Pump Inhibitor Use: A Review. Basic Clin. Pharma Tox..

[30] Joly F., Mayeur C., Messing B., Lavergne-Slove A., Cazals-Hatem D., Noordine M.L., Cherbuy C., Duée P.-H., Thomas M. (2009). Morphological adaptation with preserved proliferation/transporter content in the colon of patients with short bowel syndrome. Am. J. Physiol.-Gastrointest. Liver Physiol..

[31] Lam J.R., Schneider J.L., Zhao W., Corley D.A. (2013). Proton Pump Inhibitor and Histamine 2 Receptor Antagonist Use and Vitamin B_12_ Deficiency. JAMA.

[32] Braga C.B.M., Vannucchi H., Freire C.M.M., Marchini J.S., Júnior A.A.J., De Carvalho Da Cunha S.F. (2011). Serum Vitamins in Adult Patients With Short Bowel Syndrome Receiving Intermittent Parenteral Nutrition. J. Parenter. Enter. Nutr..

[33] Berger M.M., Amrein K., Barazzoni R., Bindels L., Bretón I., Calder P.C., Cappa S., Cuerda C., D’Amelio P., de Man A. (2024). The science of micronutrients in clinical practice—Report on the ESPEN symposium. Clin. Nutr..

[34] Pironi L., Corcos O., Forbes A., Holst M., Joly F., Jonkers C., Klek S., Lal S., Blaser A.R., Rollins K.E. (2018). Intestinal failure in adults: Recommendations from the ESPEN expert groups. Clin. Nutr..

